# Use of a 3D-printed body surface percutaneous puncture guide plate in vertebroplasty for osteoporotic vertebral compression fractures

**DOI:** 10.1371/journal.pone.0276930

**Published:** 2022-11-28

**Authors:** Jianquan Chen, Xinyuan Lin, Zhouming Lv, Maoshui Chen, Taosheng Huang

**Affiliations:** 1 The Second Clinical Medicine College, Guangzhou University of Chinese Medicine, Guangzhou, PR China; 2 Department of Orthopaedics, Guangdong Province Hospital of Traditional Chinese Medicine, Zhuhai Branch, Zhuhai, Guangdong, China; 3 The Second People’s Hospital of Xiangzhou District of Zhuhai, Guangdong, China; Ohio State University, UNITED STATES

## Abstract

**Background:**

Percutaneous vertebroplasty (PVP) has been used widely to treat osteoporotic vertebral compression fractures (OVCFs). However, it has many disadvantages, such as excessive radiation exposure, long operation times, and high cement leakage rates. This study was conducted to explore the clinical effects and safety of the use of a three-dimensional (3D)-printed body-surface guide plate to aid PVP for the treatment of OVCFs.

**Methods:**

This prospective cohort study was conducted with patients with OVCFs presenting between October 2020 and June 2021. Fifty patients underwent traditional PVP (group T) and 47 patients underwent PVP aided by 3D-printed body-surface guide plates (3D group). The following clinical and adverse events were compared between groups: the puncture positioning, puncture, fluoroscopy exposure and total operation times; changes in vertebral height and the Cobb angle after surgery relative to baseline; preoperative and postoperative visual analog scale and Oswestry disability index scores; and perioperative complications (bone cement leakage, neurological impairment, vertebral infection, and cardiopulmonary complications.

**Results:**

The puncture, adjustment, fluoroscopy, and total operation times were shorter in the 3D group than in group T. Visual analog scale and Oswestry disability index scores improved significantly after surgery, with significant differences between groups (both *p* < 0.05). At the last follow-up examination, the vertebral midline height and Cobb angle did not differ between groups. The incidence of complications was significantly lower in the 3D group than in group T (*p* < 0.05).

**Conclusion:**

The use of 3D-printed body-surface guide plates can simplify and optimize PVP, shortening the operative time, improving the success rate, reducing surgical complications, and overall improving the safety of PVP.

## Background

Osteoporotic fracture can cause acute and chronic pain, which has become an important disease endangering the health of the elderly. Osteoporotic vertebral compression fractures accounted for 15.8%. In addition, bone mineral density was generally lower in women than in men, and accelerated bone loss occurred in postmenopausal women, the female-to-male ratio was 1.6 [1]. Early due to the lack of diagnostic measures, it is easy to miss diagnosis or misdiagnosis, surgery is detected late or not at all [2]. Osteoporotic vertebral compression fractures (OVCFs) are caused by minor trauma, often during daily activities, because of reduced bone strength due to osteoporosis-related bone mass reduction and bone structure destruction. The incidence of OVCFs is increasing as the population ages. Osteoporotic vertebral compression fractures tend to occur in the T11-L2 vertebral bodies, which can cause kyphosis and pain. The permanent disability rate can reach 50%, which seriously affects the quality of life of patients [3–5]. Percutaneous vertebroplasty (PVP) has become the mainstay of OVCF treatment because of its analgesic effects, minimal surgical trauma, quick recovery, and few complications. However, it has a long learning curve due to the possibility that certain complications, particularly bone cement leakage, can occur, and it remains unpopular in primary hospitals [6, 7]. Many clinicians lack experience with PVP, which necessitates the acquisition of multiple radiographs, increasing radiation exposure. In addition, the required prone positioning for long durations burdens the respiratory and circulatory systems of elderly patients with comorbidities [8]. Precise positioning and puncture can effectively reduce complications. However, the creation of an ideal puncture channel often requires multiple attempts, increasing the complication risk. Some scholars believe that three-dimensional (3D) printing technology can improve the success rate of PVP for OVCFs [9]. However, this line of research is currently in the stage of printing model comparison.

Guide plates can facilitate intraoperative puncture and enhance the effects of PVP by reducing the numbers of punctures, puncture errors, and surgical complications. Traditional coplanar bone-surface guide plates have been used in PVP, but the fabrication of guide plates for percutaneous puncture using traditional methods is difficult because of skin deformation, changes in the receptor position, and the inability to view skin-surface markings on computed tomography (CT) images. We developed a 3D-printed external guide plate (patent no. 202010315803.1) to overcome the positioning inaccuracy resulting from the use of traditional coplanar guide plates. In this study, we compared the use of 3D-printed external guide plate–assisted PVP with the use of traditional PVP in the treatment of 100 OVCF cases.

## Methods

### Study design and population

The Ethics and Research Committee of Guangdong Hospital of Traditional Chinese Medicine, Zhuhai, China, approved this study (no. L2019-69-01). All procedures were performed according to the relevant guidelines and regulations. Informed consent to the use of medical records was obtained from all living participants.

A prospective cohort study was performed with patients attending the orthopedics departments of Guangdong Province Hospital of Traditional Chinese Medicine and The Second People’s Hospital of Xiangzhou District, Zhuhai, China. Patients with single-level OVCFs, as determined by magnetic resonance imaging (MRI), were enrolled between October 2020 and June 2021. Additional inclusion criteria were: waist and back pain aggravated by turning over or getting up and not relieved by 2 weeks of conservative treatment, with tenderness on percussion of the corresponding spinous process; a clear history of osteoporosis and T score (bone mineral density measured by dual-energy x-ray absorptiometry) < –2.5; and no evidence of preoperative spinal cord or nerve root damage in the corresponding fracture segment. The exclusion criteria were: serious spinal instability, such as extensive bone destruction in the posterior column; symptomatic neurological injury; non-OVCF condition (e.g., tumor or infectious disease); localized infection at the operative site or systemic sepsis; advanced age with poor general health or coagulation dysfunction; and severe obesity (body mass index > 30 kg/m^2^).

The patients were randomized to two age- and sex-matched groups using random numbers generated by online software (www.randomizer.org). All procedures were performed by the same group of surgeons experienced in both surgical approaches. Subjects in the 3D group underwent surgery with the use of a 3D-printed body surface guide plate for PVP. Those in group T underwent surgery with traditional free-hand PVP, the standard treatment at the participating institutions. Baseline data, including patient age, sex, occupation, and bone mineral density, were collected from the patients’ medical records.

### Surgical procedures

#### 3D group

**Fig 1:**
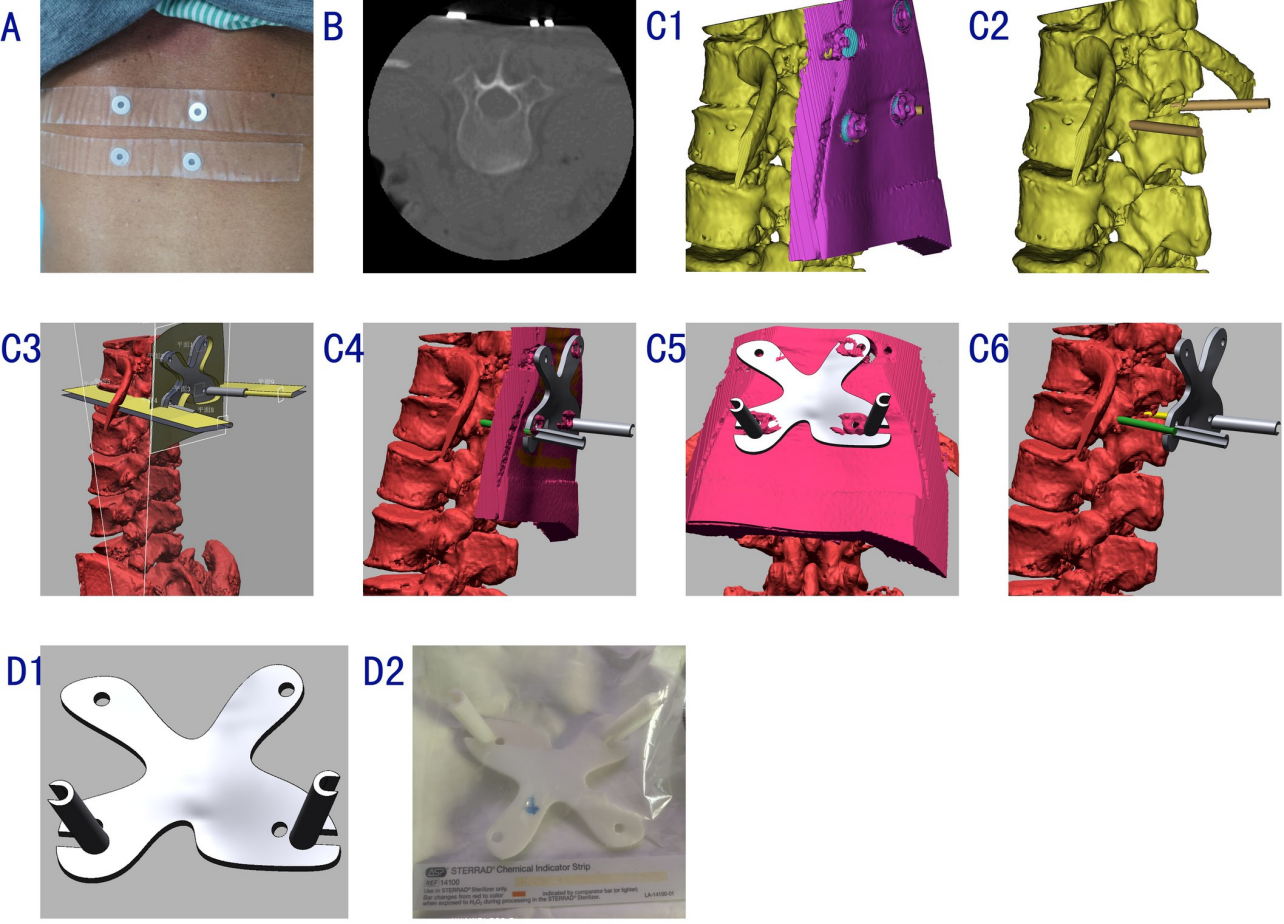
**A**: For active positioning, four or more concentric hollow metal spacers were affixed to the skin surface near the target vertebral body; for point positioning, a waterproof marker was used to mark the skin at the centers of the spacers. **B**: CT examination was performed with the patient on a supine cushion. **C1**: The vertebral model was imported from the reconstruction software in STL format. **C2**: Simulation of the working channel. **C3**: The gasket and skin model was imported in STL format for the fabrication of the coplanar guide plate and positioning of holes. **C4, 5**: Guide plate design. **D1**: The guide plate was printed with environmentally friendly PLA material at a ratio of 1:1. **D2**: The guide plate used in surgery was sterilized at low temperature.
- *3D-printed guide fabrication*. Using the CT data and 3D reconstruction software (Mimics 21.0; Materialise, Leuven, Belgium), reconstructed 3D models of the vertebrae, skin, and positioning spacers were created and imported in STL format into Design X (v. 16.02; Geomagic, Morrisville, NC, USA; Fig 1C). A body-surface puncture guide plate (1:1) was fabricated using forward and reverse engineering and 3D printing (Fig 2). The plate was sterilized before intraoperative application (Fig 1D).

- *Data acquisition*. Four or more concentric hollow metal gaskets were affixed to the skin overlying the fractured vertebra for positioning, and the center of each gasket was marked using a waterproof marker for skin tracing (Fig 1A). Thin-slice CT examination was performed using a 640-slice spiral device (Aquilion One Vision Edition; Canon Medical Systems, Otawara, Japan; slice thickness ≤ 1.0 mm, positioning accuracy correlated with section thickness) with the patient in the prone position, emulating the surgical position as much as possible, to reduce errors caused by changes in position (Fig 1B).

**Fig 2:**
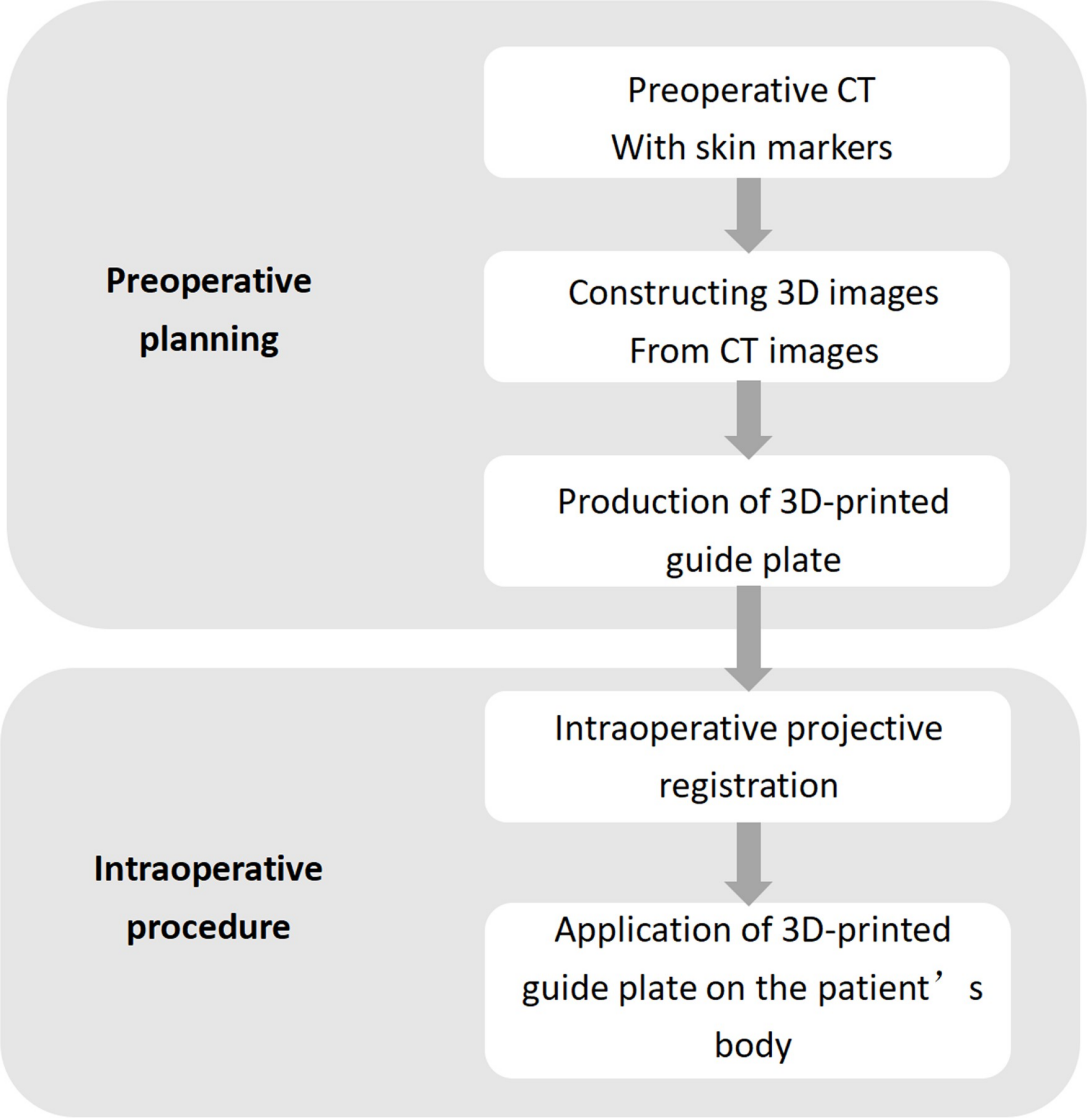
Procedure flow for the 3D group.
- *Guide plate application*. The patient was positioned prone on a radiolucent frame suitable for fluoroscopy. While the patient was relaxed, his/her skin was pressed against the guide plate, and at least four holes in the plate were aligned with the skin markings (Fig 3A).

**Fig 3:**
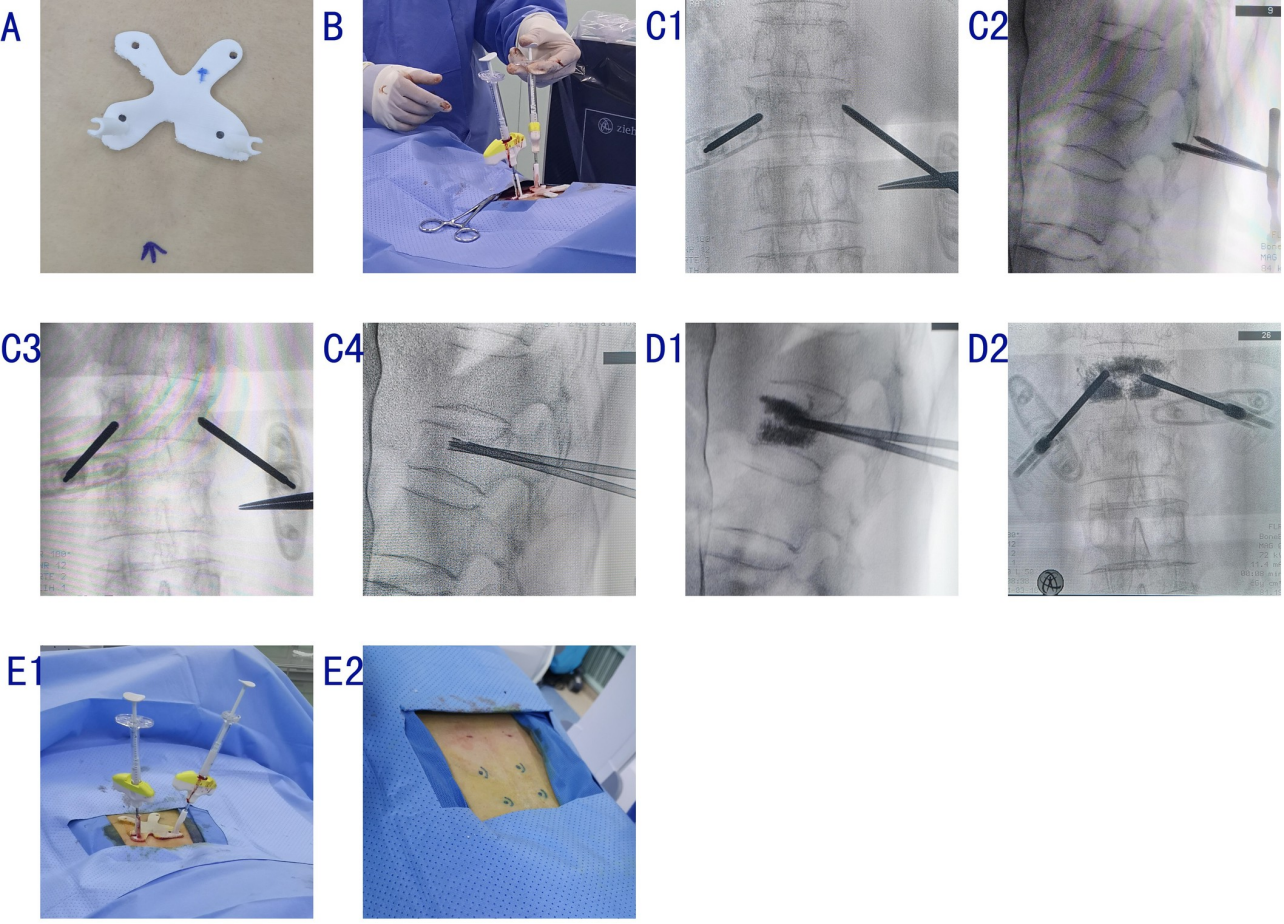
**A**: With the patient in a relaxed state, the skin was pressed against the guide and at least four holes in the guide were aligned with the skin markers. **B**: Guide plate to assist puncture and operation. **C1**: C-arm fluoroscopy was performed to determine the correct position of the puncture needle. **C2–5**: Puncture along the guide passage, with working tube placement and depth determination by fluoroscopy. **D1, 2**: Bone cement was inserted with fluoroscopic guidance. **E1**: The puncture needle was removed after the cement had solidified. **E2**: Skin glue was used to close the skin puncture site.
- *PVP*. Fluoroscopic positioning was not required once the patient’s position had been corrected using the guide plate. The guide plate was positioned, and the plate positioning holes were aligned with the skin anchor points (Fig 3B). A 10-cm-long needle was inserted through the channel indicated by the plate, and its position was confirmed via C-arm fluoroscopy, followed by 1% lidocaine injection. The needle was removed while maintaining the guide plate position. Bilateral transpedicular puncture was performed. After the posterior margin of the vertebral body had been reached, the bone needle was replaced with a working cannula (Fig 3C). When appropriate, polymethyl methacrylate (PMMA) cement was injected slowly at low pressure into the vertebral body and allowed to disperse (Fig 3D). When the cement had solidified, the cement push rod and working channel were pulled out with a single rotation, and the wound was disinfected and wrapped (Fig 3E).

#### Group T

The intraoperative puncture point was determined preoperatively using C-arm fluoroscopy, marked with a pen, and disinfected. The direction of needle insertion was determined and adjusted based on the preoperative 3D CT reconstruction and intraoperative fluoroscopy. The remaining surgical steps were identical to those performed for the 3D group.

### Postoperative management

On the second postoperative day, the patients used a brace to walk. X-rays and CT images were reviewed after surgery to assess the PMMA filling. Calcium, vitamin D, and bisphosphonates were administered routinely. Oral celecoxib (0.2 g) was administered twice daily for the first 3 postoperative days. Visual analog scale (VAS) and Oswestry disability index (ODI) scores were recorded during 1-week and 3-month follow-up visits.

### Outcome assessment

Physicians blinded to patient grouping collected all outcome data. The puncture location (per vertebra), puncture, preoperative fluoroscopy (per vertebra), fluoroscopy, and total operation times were recorded. The postoperative clinical outcomes assessed preoperatively and 7 days and 3 months postoperatively were the VAS (low back pain) and ODI (low back function) scores, midline vertebral height, and Cobb angle. The midline vertebral height (distance between the upper and lower vertebral body endplates) and Cobb angle (that between a line drawn parallel to the superior endplate of the adjacent upper vertebra and the inferior endplate of the adjacent lower vertebra) were measured on lateral X-rays.

### Statistical methods

All statistical analyses were performed using SPSS version 26.0 (IBM Corporation, Armonk, NY, USA). The distribution diagram was plotted using GraphPad Prism version 8.0.1 (GraphPad Software, San Diego, CA, USA). Categorical variables were assessed using the chi-squared and Fisher’s exact tests. Continuous variables, presented as means with SDs, were assessed using the Mann–Whitney U test and paired or unpaired *t* test with or without Welch’s correction. All statistical data are presented in tabular form. *P* < 0.05 was taken to indicate significance.

## Results

**Fig 4:**
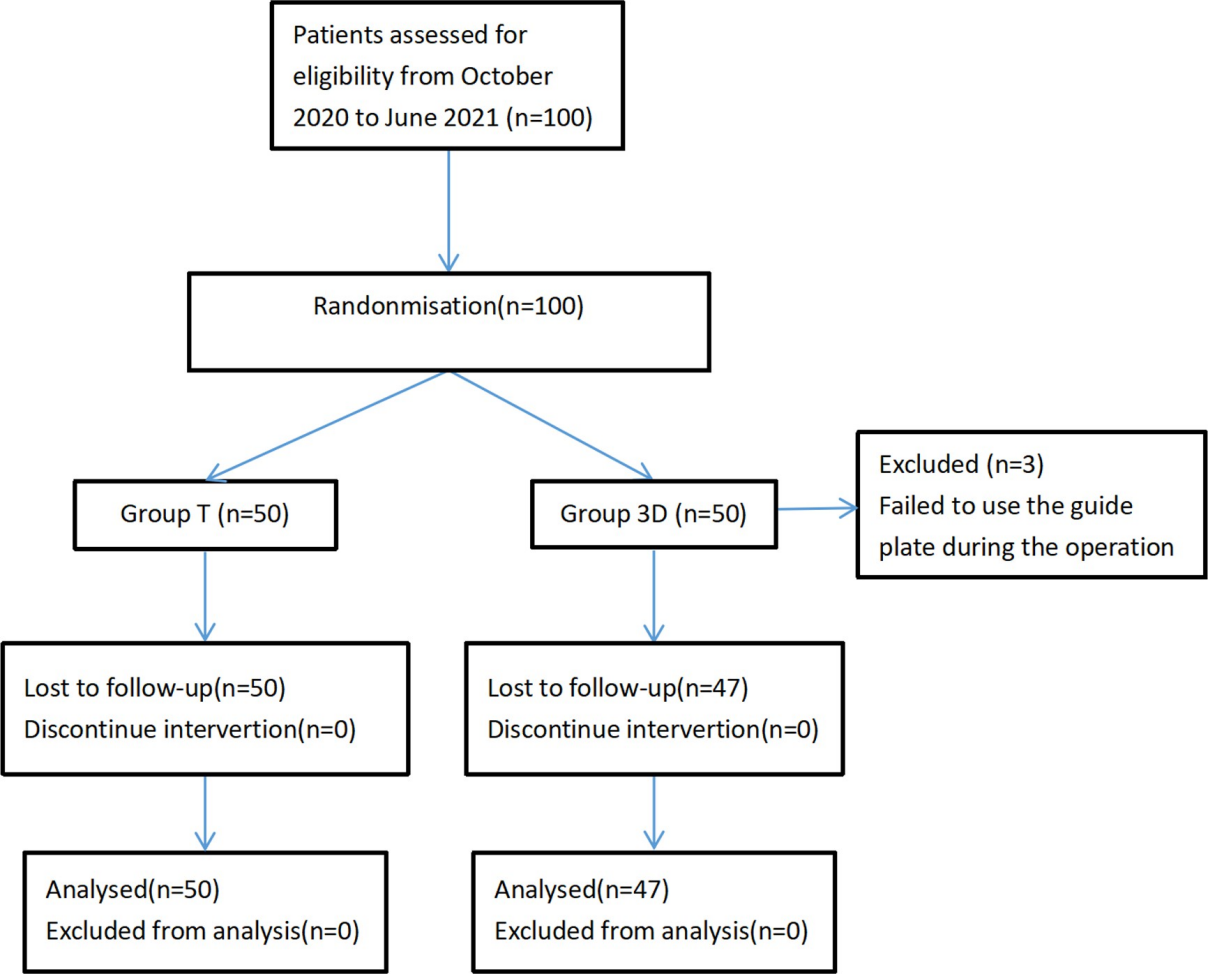
Study flow chart.

Of 100 patients with OVCFs treated at the participating centers during the study period, 97 were included in the study after screening (Fig 4). The median ages in the 3D group and group T were 79 (range, 65–90) and 80 (range, 63–91) years, respectively. In both groups, 78.4% of patients were female. The baseline characteristics of the two groups did not differ significantly (Table 1). The guide plate was not used on three patients in the 3D group, as the procedures were converted to traditional PVP intraoperatively. All times were significantly shorter in the 3D group than in group T (*p* < 0.05; Table 2).

**Table 1:** Patient characteristics.

groups	cases	Sex(cases)		Age(years)	BMD(T-score)	BMI(Kg/m^2^)	Involved segment section				
		M	F				T_10_	T_11_	T_12_	L_1_	L_2_
**Group T**	50	11	39	78.86±8.288	-3.95±0.68	24.34±3.97	12	10	14	4	10
**Group 3D**	47	10	37	80.09±6.978	-4.140±0.612	25.052±3.7235	13	8	15	4	7
**test value**		X^2^ = 0.007		t = 0.785	t = -1.417	t = 0.910	X^2^ = 0.734				
P value		0.931		0.434	0.160	0.365	0.947				
Mann whiteney U test		--		0.459	0.166	0.247	--				
P value											
Median		--		0.912	0.123	0.362	--				

M, male; F, female; BMD, bone mineral density; BMI, body mass index; T, thoracic vertebra; L, lumbar vertebra.

**Table 2:** Surgery characteristics.

Groups	Time of puncture(min)	preoperative fluoroscopy times of each vertebra	positioning puncture times of each vertebra	Time of fluoroscopy	Time of operation (min)
**Group T**	29.63±6.26	6.12±1.45	20.24±3.66	13.18±1.78	75.41±9.78
**Group 3D**	17.66±4.93	1.04±0.81	12.70±2.24	6.87±1.31	57.08±8.76
test value	-10.417	-21.106	-12.149	-19.756	-9.703
P value	0.000	0.000	0.000	0.000	0.000
Mann whiteney U test	0.000	0.000	0.000	0.000	0.000
P value					
Median	0.000	0.000	0.000	0.000	0.000

The postoperative ODI and VAS scores were significantly better than the baseline scores in both groups (*p* < 0.05; Table 3). These scores were also significantly lower at 3 months than at 7 days postoperatively (*p* < 0.05). The ODI score at 3 months was lower in the 3D group than in group T (*p* < 0.05); the VAS score did not differ significantly between groups at this timepoint. In addition, the vertebral midline height and Cobb angle at the last follow-up visit did not differ between groups (Table 4).

**Table 3:** VAS pain and ODI scores.

Groups	preoperative	Post-OP 7 day	Post-OP 3 Months
VAS pain scores			
**Group T**	7.84±1.36	4.48±1.58	1.54±0.788
**Group 3D**	8.21±1.12	3.75±1.36	1.53±0.546
test value	1.467	-2.449	-0.058
P value	0.146	0.016	0.954
Mann whiteney U test	0.134	0.010	0.548
P value			
Median	--	0.016	0.268
ODI scores			
**Group T**	39.76±5.03	30.56±5.81	22.26±5.66
**Group 3D**	41.45±4.27	25.21±6.15	16.85±6.33
test value	1.776	-4.406	-4.441
P value	0.079	0.000	0.000
Mann whiteney U test	0.089	0.000	0.000
P value			
Median	0.276	0.002	0.000

VAS, visual analog scale; ODI, Oswestry disability index.

**Table 4:** Midline vertebral heights and Cobb angles.

Groups	preoperative	Post-OP 1 day	Post-OP 3 Month
midline vertebral height			
**Group T**	18.612±3.017	19.951±3.029	19.367±3.005
**Group 3D**	18.060±2.561	19.979±2.473	19.572±2.481
test value	-0.969	0.049	0.366
P value	0.335	0.961	0.715
Mann whiteney U test	0.513	0.598	0.532
P value			
Median	0.922	0.922	0.922
Cobb angle			
**Group T**	29.249±6.674	26.276±7.049	27.597±6.832
**Group 3D**	30.544±5.537	26.346±5.753	27.265±5.688
test value	1.036	0.054	-0.259
P value	0.303	0.957	0.796
Mann whiteney U test	0.513	0.598	0.532
P value			
Median	0.912	0.912	0.922

No serious complication, such as large-vessel bleeding or permanent nerve damage, was noted. All patients’ surgical wounds healed without infection. Nerve injury was seen in one and three cases in the 3D and T groups, respectively; conservative treatment resulted in complete recovery in all cases. Vertebral collapse, peri-cement bone absorption, and infection occurred in one patient in group T with a history of rheumatoid arthritis and long-term glucocorticoid use. MRI examination suggested that spinal-canal and paravertebral abscesses had formed. Secondary surgery was performed for bone-cement removal, complete debridement, bone grafting, and internal fixation, and the infection had resolved completely at 1 year after the second surgery. Cardiopulmonary complications occurred in one case in each group. Bone-cement leakage occurred in two and six cases in the 3D and T groups, respectively. In the 3D group, the bone cement did not enter the spinal canal in any case, although it penetrated to the posterior edge of the vertebral body in one case. In group T, one case of severe bone-cement leakage at T12, with entry of the bone cement into the spinal canal and compression of the spinal cord, occurred. The patient experienced postoperative numbness and pain in both lower limbs. Emergency surgery was performed to remove the cement, after which the patient’s symptoms resolved.

## Discussion

The success rate of PVP has improved dramatically with technological advancements, but the procedure continues to be associated with certain risks. The use of 3D-printed guide plates may reduce the surgical risks and shorten the learning curve for the procedure. In recent years, 3D-printed guide plate technology has gained popularity in the field of orthopedics, as such plates facilitate accurate screw placement [10, 11].

Currently, reconstructed CT data are used to create 3D digital models of bone and other rigid surfaces for the fabrication of guide plates based on anchor point shape and anatomical characteristics. 3D-printed surgical guide plates contact the bone surface closely [12, 13] and enable accurate assessment of the position, direction, and depth of puncture during surgery [14]. The application of traditional guide plates often requires skin incision to expose certain bony surfaces, and the accuracy of plate positioning correlates strongly with the coplanar size and degree of coplanar irregularity [15, 16]. To improve accuracy, a larger incision is required to expose the bone surface or other anatomical parts close to the joint. This procedure is excessively invasive, requires structural stability and the use of nontoxic materials, and has the disadvantages of the difficulty guide-plate disinfection and sterilization [17–19]. Traditional coplanar guide plates cannot be used against soft skin surfaces, which are easily deformed and affected by changes in position [20]. Although traditional skin guide plates can be minimally invasive, their use entails the loss of positioning accuracy and universality [21]. Some researchers have reported the attachment of four bony bases (electrodes) to the lower back skin for CT reconstruction, which allows accurate positioning but requires the preservation of the lower back bases until surgery. Thus, the patient must be kept prone or lateral for 1 day to avoid the effects of pedestal displacement on accuracy [22].

We fabricated 3D-printed body-surface guide plates with active registration positioning combined with anatomical marker positioning, used four or more concentric hollow metal spacers on the body surface close to the affected vertebral body as anchor points, and then performed CT to obtain images. A combination of computer-aided design, forward and reverse modeling, and 3D printing was used. The pre-designed anchor points on the guide plate were aligned with the positioning anchor points on the skin, enabling accurate guide-plate placement through active registration. The body-surface guide plate can be used directly on the skin surface without incision, with the use of the bone surface to achieve precision. In addition, we used a simple guide plate with only four anchor points coplanar with the skin surface, which could be applied without disinfection. When the guide plate is placed to completely overlap the anchor points, it is close to the position determined by CT. This approach minimized positioning inconsistency between the CT indication and intraoperative guide plate placement. Practically, we also observed a very high degree of intraoperative positioning accuracy when the anchor point of the postural guide plate had been placed correctly, with little need for puncture adjustment through fluoroscopy. In this study, the use of CT-based positioning guide technology for PVP reduced the need for fluoroscopy, needle positioning time, radiation exposure, puncture damage, and complication risk. This technology can also reduce the operation time and number of punctures because of the high single-puncture success rate.

The three patients in the 3D group on whom the guide plate was not used due to surgical conversion were emaciated with severe skin folds, which made plate fitting difficult and led to obvious deviation of puncture angle. After several adjustments, the puncture was still not successful. In the other 47 cases, surgery with the aid of the 3D-printed body-surface guide plate was successful, with reduced puncture, adjustment, and total operation times relative to traditional PVP. All 97 surgeries were completed successfully, and final follow-up examinations revealed that the procedures had good clinical efficacy. Residual pain after PVP is related closely to cement leakage and uneven distribution, and thoracolumbar fascia injury [23]. In the 3D group, precise preoperative planning resulted in good bone cement dispersal with no clumping. In group T, precise positioning of the needle on the bone for unarmed puncture was difficult, often necessitating multiple punctures and resulting in thoracic and lumbar fascia injury, which are sources of early postoperative pain [24].

Precise puncture positioning and ensuring that the working channel allows the target to be reached are prerequisites for good bone-cement dispersion. Different pedicle heights, widths, and angles have been reported [25, 26], and no clear reference value for needle insertion in clinical practice has been established; this procedure requires experience and brings great uncertainty to the operation. In addition, conventional PVP is generally completed under C-arm fluoroscopy, and the need for repeated auxiliary adjustments during puncture increases the radiation exposure of the patient and operator [27]. Active positioning with the 3D-printed auxiliary guide plate closely follows the structure of the diseased vertebra and enables accurate reconstruction of the pedicle height and width from multiple angles. The software-based channel design enables planning for thin pedicles and those with abnormal structures, for example by selection of a fine working channel. In addition, the use of CT images for preoperative planning and precise guide-plate positioning improves the puncture accuracy and reduces the risk of bone-cement leakage. The angle of the Jamshidi needle normally does not require extensive intraoperative adjustment, merely fine tuning. The direction and angle of this needle, and the bone-cement diffusion direction, are difficult to control using the traditional puncture method. The use of the 3D-printed guide plate with accurate design of the needle entry point and angle and a safe needle entry channel enables rapid and accurate puncture, which reduces the overall operation time. With first-generation guide plates, the needle guide placed during puncture could not be removed until after the surgery, and fine adjustment of the channel was difficult. The working channel of the guide plate described here is open, a design improvement that does not affect the puncture and enables observation through the channel depth. The positioning needle is small and is placed in the center of and parallel to the working channel. The improved guide plate also features a detachable working-channel sleeve to aid the positioning of a small syringe needle.

### Study limitations

Our study sample was small, as this study was preliminary and conducted to promote the application of the guide plate; further investigation of the plate’s safety, practicality, and clinical value is needed. This study had an observational case-control design, which involves selection bias; larger multicenter randomized controlled studies are needed. In addition, the use of 3D-printed guides may not be suitable for obese or emaciated patients because of the difficulty of fitting them to the skin. Several adjustments may be needed with these patients, considering the softness of the skin and required surgical position. Minimization of the interval between CT examination and surgery or the use of additional anchor points may be useful in such cases; further investigation of these possibilities is needed. The follow-up period was short in this study, and studies with longer follow-up periods are needed to verify our result.

## Conclusions

This observational case-control study demonstrated that the use of a 3D-printed body-surface guide plate for PVP is clinically feasible. This approach can simplify and optimize the surgery, shortening the operative time, improving the success rate, reducing surgical complications and unnecessary radiation exposure, and leading to the overall improved safety of PVP.

## Supporting information

S1 Data (XLSX)

S2 Data (XLSX)

## Abbreviations

3D: three-dimensional
CT: computed tomography
MRI: magnetic resonance imaging
ODI: Oswestry disability index
OVCF: osteoporotic vertebral compression fracture
PMMA: polymethyl methacrylate
PVP: percutaneous vertebroplasty
SD: standard deviation
VAS: visual analog scale
